# A Comparison of the Immunogenicity and Safety of an Additional Heterologous versus Homologous COVID-19 Vaccination among Non-Seroconverted Immunocompromised Patients after a Two-Dose Primary Series of mRNA Vaccination: A Systematic Review and Meta-Analysis

**DOI:** 10.3390/vaccines12050468

**Published:** 2024-04-28

**Authors:** Chatchaya Nangsue, Karan Srisurapanont, Tavitiya Sudjaritruk

**Affiliations:** 1 Faculty of Medicine, Chiang Mai University, Chiang Mai 50200, Thailand; chatchaya_nangsue@cmu.ac.th (C.N.); karan_srisu@cmu.ac.th (K.S.); 2 Division of Infectious Diseases, Department of Pediatrics, Faculty of Medicine, Chiang Mai University, Chiang Mai 50200, Thailand; 3 Clinical and Molecular Epidemiology of Emerging and Re-Emerging Infectious Diseases Research Cluster, Faculty of Medicine, Chiang Mai University, Chiang Mai 50200, Thailand

**Keywords:** heterologous vaccine regimen, homologous vaccine regimen, immunocompromised patients, immunogenicity, safety, systematic review, meta-analysis, randomized controlled trial

## Abstract

This systematic review and meta-analysis aimed to compare the immunogenicity and safety of an additional heterologous (viral vector) versus homologous (mRNA) COVID-19 vaccine dose among non-seroconverted immunocompromised patients after a two-dose primary series of mRNA vaccine. We searched studies published up to 21 June 2023 in PubMed, Scopus, and Embase. The meta-analysis was conducted to compare the seropositivity rates based on anti-SARS-CoV-2 spike protein IgG (anti-S IgG) and SARS-CoV-2-specific T-cell immune response rates, assessed by interferon-γ release assay at 4 weeks, and the incidences of serious adverse events (SAEs) within 28 days between the two vaccine regimens. In four included randomized controlled trials (RCTs), there were no statistically significant differences in the seropositive rate of anti-S IgG (risk ratio [RR]: 0.79, 95% CI: 0.48–1.29) and the concentration of SARS-CoV-2 interferon-γ (RR: 1.19, 95% CI: 0.96–1.48) between heterologous and homologous regimens. The heterologous regimen exhibited a significantly lower incidence of injection pain (RR: 0.55, 95% CI: 0.45–0.69), but a higher incidence of headache (RR: 1.44, 95% CI: 1.02–2.02) compared with the homologous regimen. No vaccine-related SAEs were reported within 28 days following vaccination. An additional heterologous or homologous COVID-19 vaccine dose was well tolerated and demonstrated a comparable vaccine immunogenicity among non-seroconverted immunocompromised patients who were initially vaccinated with a two-dose COVID-19 mRNA vaccine. This finding supports the recommendations of an extended primary series of COVID-19 vaccination in immunocompromised persons.

## 1. Introduction

The wide spread of coronavirus disease 2019 (COVID-19) has inevitably afflicted endless aspects of life, importantly, human health, livelihood, society, and economics. The pandemic has currently resulted in over 774 million confirmed cases and over 7 million deaths worldwide, as reported by the World Health Organization (WHO) weekly epidemiological update on COVID-19, published on 7 January 2024 [1]. The global deleterious impacts of the COVID-19 pandemic have called for an exigent solution, which has led to the rapid discovery and investigation of a myriad of vaccines against severe acute respiratory syndrome coronavirus 2 (SARS-CoV-2), which can help reduce the risk of infection and prevent the progression to severe disease, including hospitalization and possible death related to COVID-19 [2].

At the end of December 2022, there were 50 COVID-19 vaccines that had been authorized or licensed for use in at least one country, of which 12 had been granted emergency use listing (EUL) by the WHO [3]. To date, a total of 13.5 billion doses of COVID-19 vaccine have been administered globally, and approximately 19,000 doses are administered daily. Overall, 65% of the world’s population have completed an initial COVID-19 vaccination protocol (two doses for most vaccines, one or three doses for a few manufacturers), and only 35% have received COVID-19 vaccination booster doses [4]. Although most of the available COVID-19 vaccine regimens, both homologous and heterologous prime–boost schedules, exhibit high effectiveness in reducing infection, disease severity, hospitalization, and mortality, with favorable safety and immunogenicity profiles in healthy populations [5,6,7], the vaccine-elicited immunity is not perpetual and requires enhancement by booster vaccination to ensure the sustainability of protective immunity and improve vaccine efficacy over time [8,9].

Several previous studies have demonstrated that individuals with comprised immune systems, including organ transplant recipients, patients with cancer, and patients requiring immunosuppressive treatment for an autoimmune disease, fail to demonstrate seroconversion following a primary series of COVID-19 vaccination with two doses of any WHO EUL vaccine, compared with immunocompetent individuals [10]. Additionally, based on real-world vaccine effectiveness data, immunocompromised patients exhibit reduced protection against symptomatic infection, hospitalization, and severe manifestations related to COVID-19 [11,12]. These findings highlight the importance of an extended primary series with an additional (third) dose of COVID-19 vaccine among this vulnerable population for protecting them from poor disease outcomes as the COVID-19 pandemic continues and eventually moves into an endemic state. Currently, the United States Centers for Disease Control and Prevention (US CDC) and the WHO recommend a three-dose primary COVID-19 vaccination series for mRNA vaccines in moderate to severely immunocompromised patients to enhance vaccine-elicited immune responses and raise the proportion of individuals with sufficient seroprotection against COVID-19 [13,14]. Yet, there is still a paucity of data concerning the immunogenicity and safety of an additional COVID-19 vaccine dose in these vulnerable populations, particularly those who had no seroconversion after a two-dose COVID-19 vaccination. In addition, whether a heterologous or homologous third dose vaccine schedule provides the optimal vaccine profiles remains uncertain.

This systematic review and meta-analysis aimed to compare the immunogenicity and safety profiles of an additional heterologous versus homologous COVID-19 vaccine dose among non-seroconverted immunocompromised patients who have completed a primary series of COVID-19 vaccination with two doses of mRNA vaccine. The evidence will support decision making in public health policy for the health authorities.

## 2. Materials and Methods

### 2.1. Protocol and Registration

The protocol of this systematic review and meta-analysis was registered in PROSPERO (registration ID: CRD42023381750). The study was conducted according to the Preferred Reporting Items for Systematic Reviews and Meta-Analyses (PRISMA) 2020 statement [15]. This study was granted an exemption by the Research Ethics Committee, Faculty of Medicine, Chiang Mai University (Exemption number: 0300/2566).

### 2.2. Search Strategy and Selection Criteria

We searched studies published up to 21 June 2023 in PubMed, Scopus, and Embase. The following keywords were considered to form the search strategies: ‘heterologous’, ‘homologous’, ‘immunocompromised’, ‘non-seroconverted’, ‘transplant’, ‘cancer’, ‘autoimmune’, ‘COVID-19’, ‘SARS-CoV-2’, ‘nCOV19’, ‘vaccine’, ‘booster’, and ‘random’. Some studies from previously published review articles were manually acquired.

The pre-specified inclusion criteria during screening were (i) immunocompromised patients; (ii) non-seroconverted; (iii) immunized with a primary series of two-dose mRNA vaccine; (iv) reporting of seropositivity rates of anti-SARS-CoV-2 spike protein IgG (anti-S IgG; primary outcome), and/or SARS-CoV-2-specific T-cell immune response rates (secondary outcome) and incidence of adverse events following immunization (AEFIs, secondary outcome); and (v) comparing the outcomes between an additional heterologous (viral vector) versus homologous (mRNA) COVID-19 vaccine dose. The searches and the study selection had no restrictions of language, publication date, or type of publication.

Two authors (CN and KS) independently selected studies, collected data, assessed the risk of bias, and evaluated the certainty of evidence. Discrepancies between their results were resolved by a consensus discussion or by the decision of a third author (TS).

### 2.3. Selection Process and Data Collection

Records from databases were assembled, and the abstracts and the full-text articles of each work were assessed to ascertain the eligibility of studies. The data were extracted into an electronic data record form using Zotero. The acquired data included study ID (first author, year), country, regimen of an additional COVID-19 vaccine dose, interval between the second and third dose of COVID-19 vaccine, sample size, female-to-male ratio, mean age, outcomes of interest, and information for the risk of bias assessment. Outcome data were extracted separately for patients receiving an additional heterologous and homologous COVID-19 vaccine dose. For the studies reporting the data more than once, only the most recent results were collected.

### 2.4. Endpoints

The primary outcome was the difference in the seropositivity rates, based on anti-S IgG, at 4 weeks after vaccination between participants receiving an additional heterologous (viral vector, e.g., ChAdOx1 nCoV-19 and Ad26.COV2.S) and homologous (mRNA, e.g., BNT162b2 and mRNA1273) COVID-19 vaccine dose. Since the T-cell immune response contributes to a remarkable protection against severe SARS-CoV-2 infections and is a crucial component of the adaptive immune response to COVID-19 vaccines [16,17,18], the secondary outcomes were the differences in the SARS-CoV-2-specific T-cell immune response rates, assessed by an interferon-γ release assay (e.g., ELISpot or QuantiFERON SARS-CoV-2) at 4 weeks. The secondary outcomes also included the incidence of serious adverse events (SAEs) within 28 weeks and other solicited adverse events (AEs), both local, e.g., pain at the injection site, and systemic, e.g., headache, fatigue, myalgia, and arthralgia, within 7 days following an additional vaccination between the two vaccine regimens.

### 2.5. Risk of Bias and Quality Assessment

The included studies were assessed for their risk of bias using version 2 of the Cochrane risk-of-bias tool for randomized trials (RoB-2) [19]. The bias was examined in five domains, which included (i) the randomization process, (ii) deviations from the intended interventions, (iii) missing outcome data, (iv) measurement of the outcomes, and (v) selection of the reported result.

### 2.6. Certainty of Evidence Evaluation

The certainty of the evidence was evaluated using the Grading of Recommendations Assessment, Development and Evaluation (GRADE) guidelines [20]. The domains being assessed included risk of bias, inconsistency, indirectness, imprecision, publication bias, large effect size, dose–response, and all plausible residual confounding.

### 2.7. Data Analysis

We performed the meta-analysis to compare the seropositivity rates of anti-S IgG and the SARS-CoV-2-specific T-cell immune response rates between non-seroconverted immunocompromised patients who received an additional homologous (viral vector) and heterologous (mRNA) COVID-19 vaccine dose. In addition, SAEs and commonly solicited AEs following immunization were also compared between groups of participants. The magnitude of difference was summarized with risk ratios (RRs) and 95% confidence intervals (95% CIs).

The random-effects inverse variance method was employed to pool the rates and adjust estimates across studies. The effect sizes were weighted, and the between-study variance was derived through the DerSimonian–Laird method. An *I*^2^ statistic ≥ 50% represented high heterogeneity. Publication bias was assessed by visualization of the funnel plot and quantified using Egger’s regression test. A *p*-value < 0.05 was considered statistically significant. All statistical analyses were carried out using the meta package (version 4.0.5) of R programming language (version 4.18-1) under the environment of RStudio (version 1.4.1103).

## 3. Results

### 3.1. Study Selection and Characteristics

**Figure 1:**
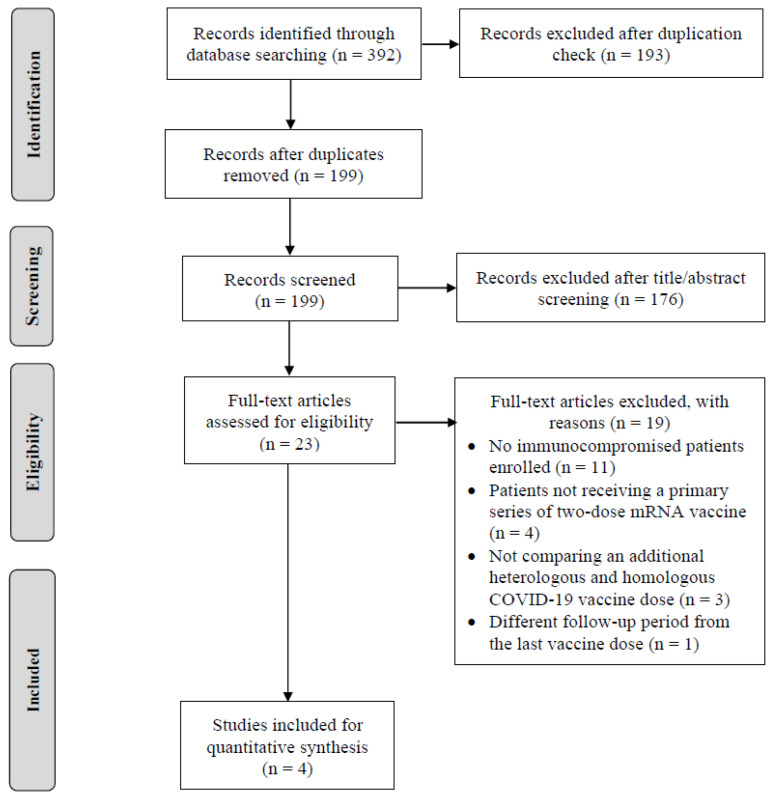
PRISMA diagram showing the process of study selection. The systematic review and meta-analysis searched three databases, removed the duplicated records, and screened the titles and abstracts of records. After examining 23 full-text articles of relevant studies, 4 studies were included in the quantitative synthesis.

In the initial literature search, 392 potential studies were identified up to 21 June 2023 (109 in PubMed, 120 in Scopus, and 163 in Embase). A total of 193 duplicates were removed, which left 199 studies for screening. After reading the titles and abstracts, 176 studies were excluded. Of the remaining 23 studies under full-text review, we further excluded 19 irrelevant studies for the following reasons: no immunocompromised patients being enrolled (*n* = 11); patients not receiving a primary series of two-dose mRNA vaccine (*n* = 4); not comparing an additional heterologous and homologous COVID-19 vaccine dose (*n* = 3); and a different follow-up period from the last vaccine dose (*n* = 1). Finally, four studies were included in this meta-analysis based on the eligibility criteria for study inclusion [21,22,23,24]. The flow chart of study selection is shown in Figure 1.

Among the four studies included in the meta-analysis, all were randomized controlled trials (RCTs). The analysis involved 444 non-seroconverted immunocompromised patients, including kidney transplant recipients (*n* = 343), patients with chronic inflammatory rheumatic or neurologic diseases under rituximab therapy (*n* = 55), and patients under immunosuppressive treatments (*n* = 46). Overall, the mean age of participants was 61.1 years, and 43% were females. In total, 220 participants received an additional heterologous (viral vector: ChAdOx1 nCoV-19 or Ad26.COV2.S) vaccine, and 224 received a homologous (mRNA: BNT162b2 or mRNA1273) COVID-19 vaccine dose after a two-dose primary series of mRNA vaccine. Among the participants receiving a heterologous vaccine regimen, the mean age was 61.1 years, and 41% were female; whereas the mean age was 61.2 years, and the female proportion was 45% for those receiving a homologous regimen. Additionally, among the studies with available data, the overall mean interval between the second and third dose of COVID-19 vaccine was 139 days, which were 140 days and 138 days for participants receiving an additional heterologous and homologous vaccine dose, respectively. The characteristics of the included studies are summarized in Table 1. Additionally, the types of immunodeficiency disorders are described in Tables S1 and S2 for participants receiving an additional heterologous and homologous COVID-19 vaccine, respectively.

### 3.2. Risk of Bias

All four RCTs included in the meta-analysis were evaluated for the risk of bias by following the instructions of the Cochrane ROB-2. All studies were considered to have a low risk of bias. Table S3 shows the results of the Cochrane ROB-2 assessment of all included studies.

### 3.3. Results of Individual Studies

At four weeks after an additional (third dose) COVID-19 vaccination, the seropositivity rates based on anti-S IgG antibodies were not significantly different between participants receiving heterologous (viral vector) and homologous vaccines (mRNA) in all studies [21,22,24] except one, which demonstrated a significantly higher seropositivity rate among the homologous group [23]. Additionally, in the studies from which data were available [21,22,24], there were no significant differences in the SARS-CoV-2-specific T-cell immune response rates, using an interferon-γ release assay, between groups of participants. For the common solicited AEs within 7 days following immunization, there was a significantly higher incidence of pain at the injection site in the homologous group [24], and significantly higher incidences of headache [24] and fatigue [21] among the heterologous group. Nevertheless, the incidences of myalgia and arthralgia were not statistically different between the participant groups [21,22,23,24]. Notably, an additional COVID-19 vaccine dose, both viral vector and mRNA, was well tolerated among the study participants. No SAEs were reported within 28 days following vaccination in any of the included studies [21,22,23,24].

### 3.4. Meta-Analytic Results

**Figure 2:**
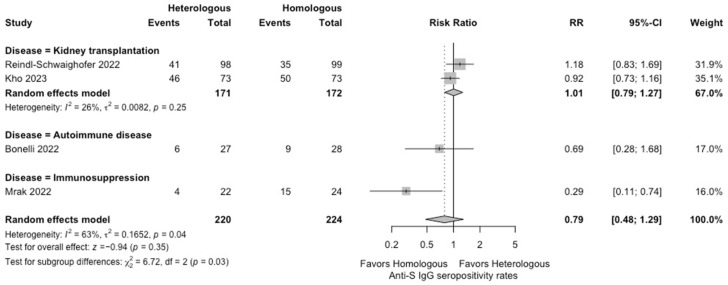
Forest plots of the meta-analysis comparing the seropositivity rates of anti-SARS-CoV-2 spike protein IgG between participants receiving an additional heterologous (viral vector) versus homologous (mRNA) COVID-19 vaccination [21,22,23,24].

**Figure 3:**
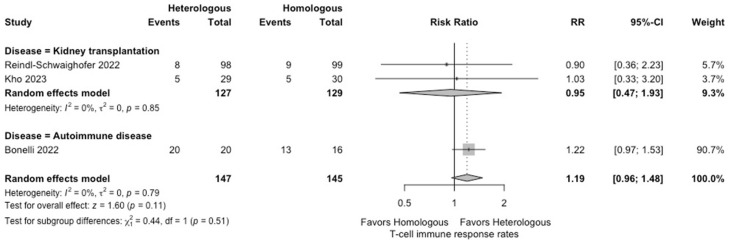
Forest plots of the meta-analysis comparing the SARS-CoV-2-specific T-cell immune response rates between participants receiving an additional heterologous (viral vector) and homologous (mRNA) COVID-19 vaccination [21,22,24].

**Figure 4:**
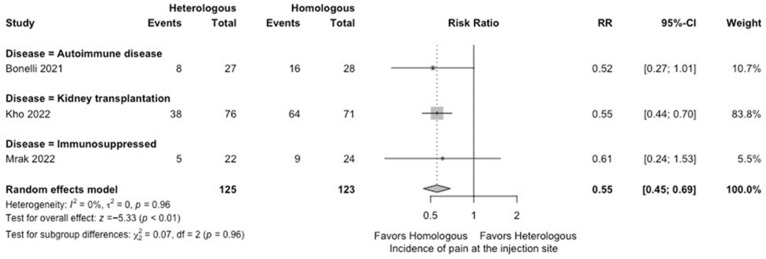
Forest plots of the meta-analysis comparing the incidence of pain at the injection site between participants receiving an additional heterologous (viral vector) and homologous (mRNA) COVID-19 vaccination [21,23,24].

**Figure 5:**
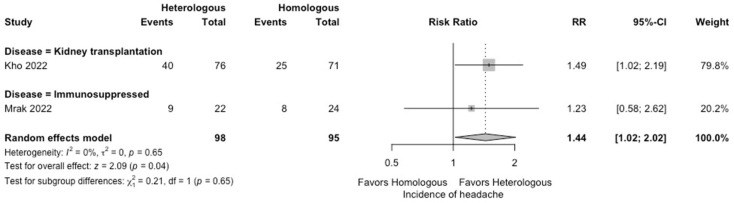
Forest plots of the meta-analysis comparing the incidence of headache between participants receiving an additional heterologous (viral vector) and homologous (mRNA) COVID-19 vaccination [23,24].

**Figure 6:**
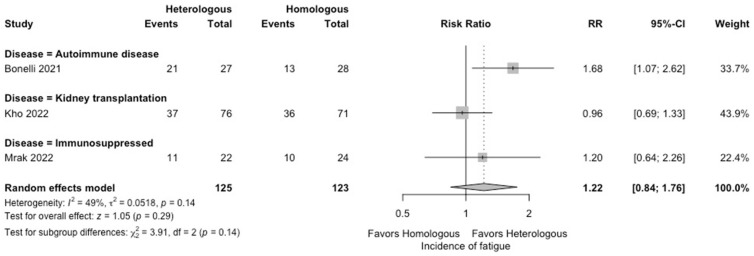
Forest plots of the meta-analysis comparing the incidence of fatigue between participants receiving an additional heterologous (viral vector) and homologous (mRNA) COVID-19 vaccination [21,23,24].

**Figure 7:**
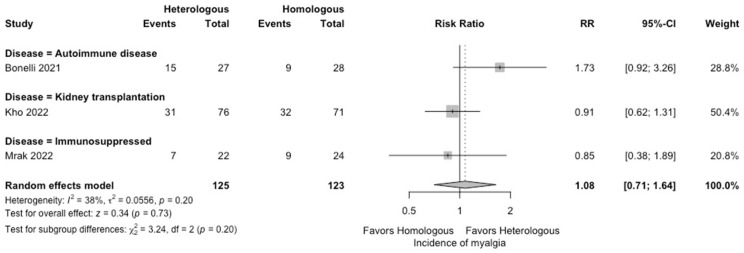
Forest plots of the meta-analysis comparing the incidence of myalgia between participants receiving an additional heterologous (viral vector) and homologous (mRNA) COVID-19 vaccination [21,23,24].

**Figure 8:**
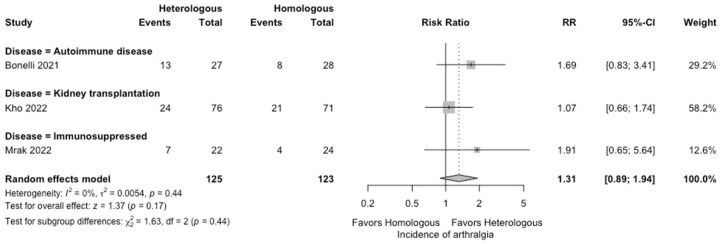
Forest plots of the meta-analysis comparing the incidence of arthralgia between participants receiving an additional heterologous (viral vector) and homologous (mRNA) COVID-19 vaccination [21,23,24].

In the meta-analysis, there were no statistically significant differences in the seropositivity rates based on anti-S IgG antibodies (four studies; RR: 0.79, 95% CI: 0.48–1.29, *I*^2^ = 63%) (Figure 2), and the SARS-CoV-2-specific T-cell immune response rates based on an interferon-γ release assay (four studies; RR: 1.19, 95% CI: 0.96–1.48, *I*^2^ = 0%) (Figure 3) between participants receiving heterologous and homologous vaccination. Within 7 days after an additional vaccination, participants in the heterologous group reported a significantly lower incidence of pain at the administration site (three studies; RR: 0.55, 95% CI: 0.45–0.69, *I*^2^ = 0%) (Figure 4), but described a significantly higher incidence of headache (two studies; RR: 1.44, 95% CI: 1.02–2.02, *I*^2^ = 0%) (Figure 5). The incidences of fatigue (three studies; RR: 1.22, 95% CI: 0.84–1.76, *I*^2^ = 49%) (Figure 6), myalgia (three studies; RR: 1.08, 95% CI: 0.71–1.64, *I*^2^ = 38%) (Figure 7), and arthralgia (three studies; RR: 1.31, 95% CI: 0.89–1.94, *I*^2^ = 0%) (Figure 8) were not significantly different between the groups of participants. Overall, no vaccine-related SAEs following an additional COVID-19 vaccine dose were found within 28 days after vaccination.

### 3.5. Publication Bias

Figure S1 shows the funnel plots of a meta-analysis comparing the outcomes of interests between participants who received an additional heterologous versus homologous COVID-19 vaccination. By visualization, all funnel plots did not show any significant asymmetry data. Egger’s regression test was not applied, since there were less than 10 included studies in this review.

### 3.6. Certainty of Evidence

The certainty level of the meta-analytic result for each outcome of interest, including the anti-S IgG seropositivity rate; SARS-CoV-2-specific T-cell immune response rate; and AEFIs, e.g., pain at the injection site, headache, fatigue, myalgia, and arthralgia, was initially high, because all included studies were RCTs. However, since the quantitative results of publication bias were not available, the certainty levels of the meta-analytic results for all outcomes were downgraded by one level. In addition, since there was high heterogeneity of the anti-S IgG seropositivity rates across the included studies (*I*^2^ = 63%), the certainty level of the meta-analytic result for this outcome was downgraded by one level. Furthermore, the certainty levels of the meta-analytic results for anti-S IgG seropositivity rates and SARS-CoV-2-specific T-cell immune response rates were downgraded by one level as a result of imprecision (the 95% CIs overlapped the null effect). The remaining domains, including risk of bias, indirectness, large effect size, dose–response gradient, and all plausible residual confounding, were neutral and had no rating up or down. In summary, the overall certainty levels of the anti-S IgG seropositivity rate and SARS-CoV-2-specific T-cell immune response rate were summarized as very low and low, respectively, whereas the certainty levels of the other outcomes were moderate (Table S4).

## 4. Discussion

This systematic review comprises four RCTs comparing an additional heterologous (viral vector) versus homologous (mRNA) COVID-19 vaccination among non-seroconverted immunocompromised patients, including kidney transplant recipients, patients with autoimmune disorders, and patients under immunosuppressive therapy, who had completed a two-dose primary COVID-19 mRNA vaccine series. At four weeks following the third vaccination, the pooled results demonstrated that vaccine immunogenicity, based on anti-S IgG seropositivity rates and SARS-CoV-2-specific T-cell immune response rates, was comparable between the two regimens. Although participants receiving an additional heterologous vaccine dose reported a significantly higher incidence of headache and lower incidence of injection site pain compared with homologous vaccination, the other solicited local and systemic AEs were comparable. Importantly, both vaccine regimens were not related to any SAEs occurring within 28 days following vaccination among these immunocompromised patients.

Immunocompromised patients or patients with suppression of the immune system due to underlying medical conditions, such as malignancy, autoimmune disease, and being post-transplantation, or specific treatment regimens, such as immunosuppressive and immunomodulatory drugs, are at greater risk of severe manifestations, prolonged hospitalization, intensive care unit (ICU) admission, and mortality related to COVID-19 compared to the general population [25,26]. Thus, these vulnerable people are recommended to be a high-priority user group for COVID-19 vaccination according to the WHO recommendations [14,27]. Nevertheless, a weak or suboptimal immune response after vaccination with a two-dose primary series of COVID-19 vaccine are observed in this critical group of patients, particularly organ transplant recipients [10,28]. Hence, even vaccinated, these individuals are still at an increased risk of hospitalization, ICU admission, and mortality related to COVID-19 [29]. This highlights the importance of an extended primary series with an additional (third) dose of COVID-19 vaccine for this vulnerable population. Although most of the earlier pivotal COVID-19 vaccine trials excluded immunocompromised groups, the recent RCTs and large observational studies have subsequently begun to shed light on the benefits of an extra dose of COVID-19 vaccine among this population [21,22,23,24,30,31]. Yet, an appropriate vaccine regimen for a third primary dose has been controversial.

Among non-seroconverted immunocompromised patients who had completed a two-dose primary COVID-19 mRNA vaccine series, our pooled results showed that an additional heterologous (viral vector) and homologous (mRNA) COVID-19 vaccination elicited a comparable vaccine immunogenicity, regarding humoral- and cell-mediated immune responses. Likewise, a small German prospective cohort study among 25 kidney transplant recipients (median age 60 years) who were seronegative after initial COVID-19 vaccination with two-dose mRNA vaccine revealed that the humoral-mediated (e.g., anti-S IgG and vaccination-specific B cell responses) and cellular-mediated immune responses (e.g., vaccination-specific T cell responses) after a third dose of viral vector (ChAdOx1 nCoV-19; *n* = 11) and mRNA (BNT162b2; *n* = 14) were not significantly different [32]. Although one of the included RCTs demonstrated a significantly lower humoral immune response among individuals receiving a heterologous vaccine regimen, this might be attributed to the heterogeneity of the cohort of participants under immunosuppressive treatments who were enrolled in the study, as underlying disease and specific medication can contribute to a variation in the immune response to vaccination. Additionally, the small number of participants in each vaccine group might bias the comparisons [23].

An additional COVID-19 vaccine dose, either viral vector or mRNA, was safe and well-tolerated among immunocompromised participants in this study. No vaccine-related SAEs following a third dose of vaccination with both vaccine regimens were reported. Regarding the solicited AEs, our meta-analysis results showed that a homologous regimen with mRNA vaccine was associated with a higher incidence of injection site pain, whereas a heterologous regimen with viral vector vaccine was related to a higher incidence of headache for 7 days following vaccination. Similar to immunocompetent individuals who initially received two doses of BNT162b2 vaccine, transient pain at the site of intramuscular injection was the most prevalent solicited local AE of COVID-19 mRNA vaccine, both BNT162b2 and mRNA-1273, after the third-dose vaccination [33,34]. The underlying mechanism of this AE might be related to transient fasciitis of the deltoid muscles in vaccine recipients [35]. In addition, the COVID-19 viral vector vaccine was commonly found to be associated with acute headache as a solicited systemic AE, particularly among those with pre-existing primary headache. The post-vaccination headache phenotype is usually characterized by moderate-to-severe dull pain or pressure in the head, accompanied by fatigue, chills, exhaustion, photophobia, or phonophobia [36,37]. This condition could be related to the production of inflammatory mediators such as type Iβ interferon, which occurs during an immunity activation process by vaccine [36,37].

The findings of our meta-analysis emphasized the importance of an extended primary series with an additional vaccine dose for COVID-19 vaccination in immunocompromised persons. Yet, this study contains several limitations. First, a small number of eligible studies and sample size limited our ability to conduct a subgroup analysis and meta-regression analysis. Second, since all four original studies included in the meta-analysis were conducted in Western European countries [21,22,23,24], three of which were in Austria [21,22,23], the representativeness of our results may be affected by the race, ethnicity, and genetic and other physical characteristics of the included participants, as well as the SARS-CoV-2 epidemic situation and type of COVID-19 vaccine recommendations of the countries, resulting in a limited generalizability of our findings. In addition, most of the included immunocompromised patients in our meta-analysis were kidney transplant recipients (77%), who usually have more severe immunosuppression compared with other immunocompromised conditions, and the analysis did not include hematopoietic stem cell transplant recipients or cancer patients; therefore, our findings might not be applicable to wider immunocompromised patients, who might exhibit differences in vaccination responses and vaccine reactogenicity. Also, since most of the participants in this study were older adults and elderly individuals (mean age 61 years), our results might not represent findings in pediatric and young-adult populations. The heterologous vaccine data in this study were limited, as they do not include other platforms of COVID-19 vaccine, e.g., inactivated virus or protein subunit vaccine. Furthermore, the impact of the dosing interval on the magnitude of an immune response to a vaccine could not be evaluated, because the interval between the second and third COVID-19 vaccine dose were not available in the two included studies [21,23]. Importantly, since all included RCTs were conducted before the emergence of the SARS-CoV-2 omicron variant, the immunogenicity to the first generation of the COVID-19 vaccine noted in our meta-analysis might not describe the vaccine immune responses against omicron and later emerging variants well. Also, the neutralizing antibody to SARS-CoV-2, which is an important immunological surrogate endpoint to define protective antibodies against COVID-19, as well as the efficacy and effectiveness of an extended primary series of COVID-19 vaccination against severe COVID-19 illness, hospitalization, ICU admission, and mortality, were not evaluated in this study. The certainty of evidence in our estimates for vaccine immunogenicity was very low to low (Table S4); thus, future studies might change the estimates. Lastly, a further study which includes the latest publications is warranted to confirm our findings.

## 5. Conclusions

An additional COVID-19 vaccination, with either a heterologous or homologous regimen, was safe and immunogenic among immunocompromised patients who had no seroconversion after a two-dose primary series of COVID-19 mRNA vaccine. The heterologous and homologous vaccine regimen demonstrated comparable humoral and cellular immune responses. No SAEs related to vaccination were reported. These findings support the WHO recommendation of an extended primary series with an additional vaccine dose for COVID-19 vaccination among populations with a compromised immune system. A long-term follow-up study to determine the sustainability of immune responses to a third-dose vaccine, as well as the importance of a routine booster vaccination among this vulnerable population, is important for further investigations.

## Figures and Tables

**Table 1:** Characteristics of the included studies.

Study (Country)	Study Participants	Regimen of Primary Series of 2-Dose mRNA COVID-19 Vaccine	Participant Characteristics				Interval between 2nd and 3rd Dose of COVID-19 Vaccine *, Day (SD)		Regimen of an Additional COVID-19 Vaccine Dose (3rd Dose)	
			Number of Participants (F/M)		Mean Age *, Year (SD)					
			Het	Hom	Het	Hom	Het	Hom	Het	Hom
Bonelli 2022 (Austria) [21]	Patients with chronic inflammatory rheumatic or neurologic diseases under rituximab therapy	BNT162b2 or mRNA-1273	27 (18/9)	28 (23/5)	60.9 (15.0)	58.9 (18.4)	NA	NA	ChAdOx1 nCoV-19	BNT162b2 or mRNA-1273
Reindl 2022 (Austria) [22]	Kidney transplant recipients	BNT162b2 or mRNA-1273	98 (40/58)	99 (42/57)	61.2 (11.8)	61.2 (13.1)	82 (24.4)	78 (23.0)	Ad26.COV2.S	BNT162b2 or mRNA-1273
Mrak 2022 (Austria) [23]	Patients under immunosuppressive therapy	BNT162b2 or mRNA-1273	22 (7/15)	24 (10/14)	61.2 (14.9)	63.4 (11.4)	NA	NA	ChAdOx1 nCoV-19	BNT162b2 or mRNA-1273
Kho 2023 (The Netherland) [24]	Kidney transplant recipients	BNT162b2 or mRNA-1273, or a combination of both	73 (25/48)	73 (25/48)	60.1 (12.4)	57.3 (13.5)	198 (19.3)	198 (11.9)	Ad26.COV2.S	mRNA1273

Abbreviations: Ad26.COV2.S, Janssen COVID-19 vaccine; BNT162b2, Pfizer-BioNTech COVID-19 vaccine; F, female; Het, an additional heterologous COVID-19 vaccine; Hom, an additional homologous COVID-19 vaccine; M, male; mRNA-1273, Moderna COVID-19 vaccine; mRNA, messenger RNA; NA, not available. * Data are presented as mean (standard deviation, SD).

## Data Availability

The data that were used and/or analyzed are available upon request from the corresponding author.

## Supplementary Materials

The following supporting information can be downloaded at https://www.mdpi.com/article/10.3390/vaccines12050468/s1: Figure S1: Funnel plots of a meta-analysis comparing the outcomes of interests between participants who received an additional heterologous (viral vector) and homologous (mRNA) COVID-19 vaccination; Table S1: Types of immunodeficiency disorders among participants receiving an additional heterologous (viral vector) COVID-19 vaccine; Table S2: Types of immunodeficiency disorders among participants receiving an additional homologous (mRNA) COVID-19 vaccine; Table S3: Risk of bias of the included studies, assessed using Cochrane RoB-2 tool; Table S4: Certainty of evidence of the outcomes of interests, assessed using the Grading of Recommendations Assessment, Development and Evaluation (GRADE) approach.
