# Supplementary material for: A Comparison of the Immunogenicity and Safety of an Additional Heterologous versus Homologous COVID-19 Vaccination among Non-Seroconverted Immunocompromised Patients after a Two-Dose Primary Series of mRNA Vaccination: A Systematic Review and Meta-Analysis
Source: Vaccines (Basel). 2024 Apr 28;12(5):468. doi: 10.3390/vaccines12050468 (PMC11125606; doi:10.3390/vaccines12050468)
Supplement: Supplementary file 1 [file vaccines-12-00468-s001.zip › Supplementary Figure.pdf]

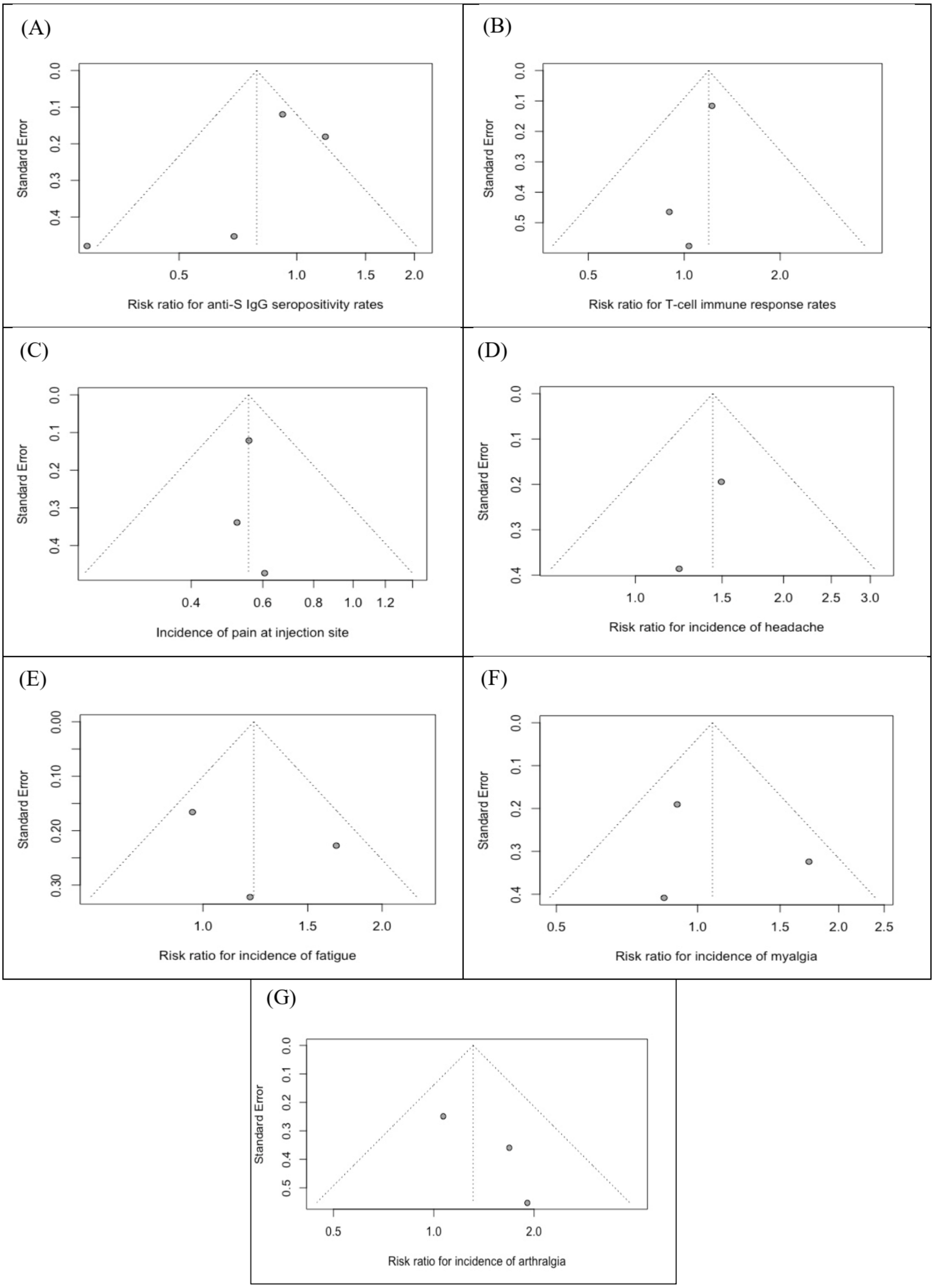

Figure S1. Funnel plots of a meta-analysis comparing the outcomes of interests between participants who received an additional heterologous (viral vector) and homologous (mRNA) COVID-19 vaccination. (A) anti-SARS-CoV-2 spike protein (anti-S) IgG seropositivity rates; (B) SARS-CoV-2-specific T-cell immune response rates; (C) incidence of pain at the injection site; (D) incidence of headache; (E) incidence of fatigue; (F) incidence of myalgia; and (G) incidence of arthralgia.
