# Supplementary material for: A Comparison of the Immunogenicity and Safety of an Additional Heterologous versus Homologous COVID-19 Vaccination among Non-Seroconverted Immunocompromised Patients after a Two-Dose Primary Series of mRNA Vaccination: A Systematic Review and Meta-Analysis
Source: Vaccines (Basel). 2024 Apr 28;12(5):468. doi: 10.3390/vaccines12050468 (PMC11125606; doi:10.3390/vaccines12050468)
Supplement: Supplementary file 1 [file vaccines-12-00468-s001.zip › Supplementary Tables.pdf]

Table S1. Types of immunodeficiency disorders among participants receiving an additional heterologous (viral vector) COVID-19 vaccine.

Study	Diagnoses														
	**AIH**	**Arthritis**	**CTD**	**HTX**	**HTX+ MM**	**IgG4-related disease**	**KTX**	**LiTX**	**LiTX+KTX**	**LuTX**	**MM**	**MS**	**RCC**	**Vasculitis**	**Breast cancer**	**Pemphigus vulgaris**
Bonelli 2022	0 (0)	11 (40.7)	7 (25.9)	0 (0)	0 (0)	2 (7.4)	0 (0)	0 (0)	0 (0)	0 (0)	0 (0)	3 (11.1)	0 (0)	4 (14.8)	0 (0)	0 (0)
Reindl 2022	0 (0)	0 (0)	0 (0)	0 (0)	0 (0)	0 (0)	98 (100)	0 (0)	0 (0)	0 (0)	0 (0)	0 (0)	0 (0)	0 (0)	0 (0)	0 (0)
Mrak 2022	0 (0)	0 (0)	1 (4.5)	10 (45.5)	0 (0)	0 (0)	0 (0)	3 (13.6)	0 (0)	6 (27.3)	1 (4.5)	0 (0)	1 (4.5)	0 (0)	0 (0)	0 (0)
Kho 2023	0 (0)	0 (0)	0 (0)	0 (0)	0 (0)	0 (0)	73 (100)	0 (0)	0 (0)	0 (0)	0 (0)	0 (0)	0 (0)	0 (0)	0 (0)	0 (0)
**Total**	**0**	**11**	**8**	**10**	**0**	**2**	**171**	**3**	**0**	**6**	**1**	**3**	**1**	**4**	**0**	**0**

Abbreviations: AIH, autoimmune hepatitis; CTD, connective tissue disease; HTX, heart transplant; HTX+MM, heart transplant and multiple myeloma; KTX, kidney transplant; LiTX= liver transplant, LiTX+KTX, liver transplant and kidney transplant; LuTX= lung transplant; MM, multiple myeloma; MS, multiple sclerosis; RCC, renal cell carcinoma.

Table S2. Types of immunodeficiency disorders among participants receiving an additional homologous (mRNA) COVID-19 vaccine.

**Study**	**Diagnoses**															
	**AIH**	**Arthritis**	**CTD**	**HTX**	**HTX+ MM**	**IgG4-related disease**	**KTX**	**LiTX**	**LiTX+KTX**	**LuTX**	**MM**	**MS**	**RCC**	**Vasculitis**	**Breast cancer**	**Pemphigus vulgaris**
Bonelli 2022	0 (0)	10 (35.7)	9 (32.1)	0 (0)	0 (0)	2 (7.1)	0 (0)	0 (0)	0 (0)	0 (0)	0 (0)	3 (10.7)	0 (0)	4 (14.3)	0 (0)	0 (0)
Reindl 2022	0 (0)	0 (0)	0 (0)	0 (0)	0 (0)	0 (0)	99 (100)	0 (0)	0 (0)	0 (0)	0 (0)	0 (0)	0 (0)	0 (0)	0 (0)	0 (0)
Mrak 2023	1 (4.2)	0 (0)	1 (4.2)	8 (33.3)	1 (4.2)	0 (0)	0 (0)	2 (8.3)	1 (4.2)	5 (20.8)	1 (4.2)	2 (8.3)	0 (0)	0 (0)	1 (4.2)	1 (4.2)
Kho 2023	0 (0)	0 (0)	0 (0)	0 (0)	0 (0)	0 (0)	73 (100)	0 (0)	0 (0)	0 (0)	0 (0)	0 (0)	0 (0)	0 (0)	0 (0)	0 (0)
**Total**	**1**	**10**	**10**	**8**	**1**	**2**	**172**	**2**	**1**	**5**	**1**	**5**	**0**	**4**	**1**	**1**

Abbreviations: AIH, autoimmune hepatitis; CTD, connective tissue disease; HTX, heart transplant; HTX+MM, heart transplant and multiple myeloma; KTX, kidney transplant; LiTX= liver transplant, LiTX+KTX, liver transplant and kidney transplant; LuTX= lung transplant; MM, multiple myeloma; MS, multiple sclerosis; RCC, renal cell carcinoma.

Table S3. Risk of bias of the included studies, assessed using Cochrane RoB-2 tool.

Study	Randomization process	Deviations from the intended interventions	Missing outcome data	Measurement of the outcome	Selection of the reported result	Overall risk of bias
Bonelli 2022	Low risk	Low risk	Low risk	Low risk	Low risk	Low risk
Reindl 2022	Low risk	Low risk	Low risk	Low risk	Low risk	Low risk
Mrak 2022	Low risk	Low risk	Low risk	Low risk	Low risk	Low risk
Kho 2023	Low risk	Low risk	Low risk	Low risk	Low risk	Low risk

Table S4. Certainty of evidence of the outcomes of interests, assessed using the Grading of Recommendations Assessment, Development and Evaluation (GRADE) approach.

**Outcome**	**Initial certainty of evidence**	**Domain**								**Overall certainty of evidence**
		**Lower if**					**Higher if**			
		**Risk of bias**	**Inconsistency**	**Indirectness**	**Imprecision**	**Publication bias**	**Large effect size**	**Dose-response gradient**	**All plausible residual confounding**	
Anti-S IgG seropositivity rate	High (RCTs)	0 (no evidence)	-1 (I^2 = 63%)	0 (no evidence)	-1 (95% CI overlapped the null effect)	-1 (no quantitative results of publication bias)	0 (no evidence)	0 (no evidence)	0 (no evidence)	Very low
SARS-CoV-2-specific T-cell immune response rate			0 (I^2 = 0%)		-1 (95% CI overlapped the null effect)					Low
Pain at injection site			0 (I^2 = 0%)		0 (no evidence)					Moderate
Headache			0 (I^2 = 0%)		0 (no evidence)					Moderate
Fatigue			0 (I^2 = 49%)		0 (no evidence)					Moderate
Myalgia			0 (I^2 = 38%)		0 (no evidence)					Moderate
Arthralgia			0 (I^2 = 0%)		0 (no evidence)					Moderate
